# The Fourth International Dental Congress

**Published:** 1904-08

**Authors:** Edward C. Kirk

**Affiliations:** Philadelphia


### THE FOURTH INTERNATIONAL DENTAL CONGRESS.[1]

BY EDWARD C. KIRK, D.D.S., SC.D., PHILADELPHIA.

What first appeals to us in the consideration of this Congress is the magnitude of its several features and the extent of the interest which the world of dentistry is manifesting in the outcome of the meeting.

There has been called to meet in St. Louis next month a gathering of dentists representing every civilized country on the globe. Each nation having any importance from the stand-point of professional dentistry will be represented by its practitioners and will contribute to the programme the latest development of its dental

[1] Abstract of a paper read before the Pennsylvania State Dental Society at Wilkes-Barre, Pa., July 13, 1904.

progress, so that as a whole the St. Louis Congress will constitute an exhibit of the world's dentistry at this dawn of the twentieth century.

To prepare for this great event has been the work assigned to a special committee appointed by the National Dental Association at its meeting in 1902 at Niagara Falls, and confirmed by the Directory of Congresses of the St. Louis Exposition in 1903.

Notwithstanding the comparatively limited time at command since the inception of the movement, the work of organization has proceeded rapidly and upon systematic lines. Committees of publicity and propaganda have been formed in every important country of Europe, as well as in Australia and Japan, so that twenty or more nations are now concerned in this movement and are actively spreading an interest and securing support in every way for the meeting. Contributions to all departments of the programme are rapidly coming in, and to an extent that is causing no small difficulty in regard to their disposal. Each State in our Union is organized by the appointment of State chairmen, who, working with their local committees, are collectively preparing the part which the United States is to perform in this dental congress of all nations.

While it is not possible to give exact figures, it may be safely stated that not less than fifteen hundred committeemen are actively at work throughout the world in preparing for the events which will together constitute the Fourth International Dental Congress. Upward of three hundred clinical demonstrations are being provided for. The ten sectional divisions of the Congress which will hold simultaneous meetings will produce not less than one hundred to one hundred and fifty essays by the leaders of dental thought in all countries, covering all departments of our profession. Dental education, legislation, history, and nomenclature will be the subjects of comprehensive reports made by experts who have for years made these subjects a specialty.

The dealers' and manufacturers' exhibit of dental supplies now includes a larger catalogue of individual exhibits than has heretofore appeared in connection with any dental meeting.

As to the probable number of members who will participate, the indications now point to a larger paid membership than has ever before attended an international dental congress.

The provisions being made by the local Committee of Arrangements for the care and entertainment of members are upon a scale which assures the success of the social features of the Congress,—a feature which, after all, is the most important civilizing influence in our dental meetings, and one which in a great international gathering contributes the most towards harmonizing the different national ideals, thus aiding the progress of dentistry as a whole.

To those in position to watch the growth of this world movement of our profession towards a common object the view is an inspiring one. The conception of our calling derived from a local environment at once loses its significance in the broader aspect which the international feature of this congress presents for consideration. In our local, State, and national associations we meet with differences of opinion as to theory and practice, and out of the multitude of counsel we extract the grist of that wisdom which becomes in time our accepted standard of practice; yet this diversity of opinion is among men similarly trained, speaking the same mother tongue, having the same patriotic sentiments and similar professional ideals. In an international congress are brought together the products of the most diverse systems of training, with corresponding differences of method and theory and the added diversity of language and national characteristics which act as modifying influences in shaping the professional tendencies of each nation towards its own standards and aims. Yet notwithstanding these characteristic national differences, there remains the one professional ideal as the feature common to dentistry in all nations,—the salvation of the human denture and the one animating desire to solve the problems of our calling upon an independent professional basis.

The attitude of mind of our professional organization, if I may so express it, has needed the developing influence of experience to so ripen it that a clearer conception of the position of dentistry among the beneficent callings of mankind might be formulated, and during the past twenty years the needed experience has come with its fruit of ripened judgment as a consequence.

In 1889 France issued a call for the First International Dental Congress. Previous to this the seeds of internationalism in dental associative work were sown by the establishment of a dental section at the International Medical Congress held in London in 1881, and repeated at the International Congress in Philadelphia in 1887. At these meetings the leaders of dentistry of the several nations were brought into contact, and there was born the realization that while all were striving for a common ideal the methods of each were widely different, and that much good would result from a more intimate specialized association that would give opportunity for the comparison and discussion of ideas upon an international and purely dental basis.

The complexity which the international features of the dental professional problem presented were such as could best be worked out upon a basis quite separate from the medical relationship, hence an international congress of dentists dealing exclusively with dentistry was determined upon; and it was France, whose leaders of dental thought were most deeply impressed with this idea, that took the initiative.

The Dental Congress of 1889, held in Paris, was an abundant success, which revealed the fact that the sentiment was strongly in favor of the independence of dentistry as a profession. This first purely dental congress international in scope accomplished the still more important result of practically demonstrating the good which might flow to all concerned by periodically bringing together in harmonious relationship the dental representatives of all nations for the discussion of those problems which are vital to the progress and success of dentistry as a profession throughout the world. In 1893 the Second International Dental Congress was held in Chicago, as the World's Columbian Dental Congress, and the third of these reunions was held in Paris in 1900. Each subsequent congress exceeded its predecessor in importance both as to magnitude of membership, output of work, and general character of results. Especially there should be noted in this connection the growth of the spirit of internationalism in dentistry with each succeeding congress. The active and leading men of all nations have on each occasion been brought into close personal contact, and have learned to regard with respect the efforts which all are making to develop those factors which in each country are placing dentistry upon a higher scientific and social plane.

That type of self-sufficiency and conceit begotten of ignorance which offends decency with its blatant claim of superiority over all others has been made unpopular by these great international meetings, and that higher and nobler spirit of according honor and praise to him who is deserving thereof regardless of his nativity or the language in which he expresses his thought is becoming the dominating principle in these associations; all of which is as it should be.

Dentistry has outgrown its swaddling clothes and is now in the period of a strong and lusty adolescence. It has grown into that independence to which it was from the first destined by virtue of its inherent usefulness to humanity. But it has grown otherwise. In the reaching outward of its spheres of influence it has escaped the geographical bounds which limit the activities of nations, and finding sympathetic response in the touch of the dental professional spirit in other nations has laid the first foundations for the formation of a dental world power which shall know no national limitations, but which shall regard our calling as of that higher order of truth and knowledge that is the exclusive possession of no country, though native it may be to some, yet, withal, a citizen of the world.

It will be seen that the international dental congress fulfils a function with which our State and national organizations are not directly concerned nor are they competent to deal.

We discuss questions of education, legislation, and reciprocity, but in their international aspects these matters develop a much wider significance. Our national pride and our patriotism are touched unpleasantly when, for example, Germany enacts legislation excluding the American graduate from using his doctor title while practising within her territory; yet in all justice there is something to be said in defence of this attitude upon the part of our transatlantic neighbor, and it is the function and purpose of the international congress to develop the kind of attitude and produce the evidence that in the course of time will set such matters straight.

The international congress movement is a movement towards harmony, by comparison and discussion of those conflicting views which interfere with dental progress and bring about a readjustment of relations upon the basis of greatest advantage to our profession throughout the world.

Much has already been accomplished. The spirit of internationalism in dentistry has taken root and is rapidly spreading its influence among the nations, so that already the fruits of its harmonizing tendency are apparent.

Not the least important step which has been taken towards conserving and developing this international spirit of confraternity among dental practitioners the world over is the creation at the Paris Congress of the Fédération Dentaire Internationale, an organization of all the delegates representing the several nations at the Congress of 1900. The purposes of this Federation are to foster the objects for which international congresses are held,—to promote and assist all movements which in an international way can contribute to the advancement of the profession of dentistry. The Federation is represented by an Executive Council, which has power to act upon behalf of the Federation as an *ad interim* committee, to accept invitations to hold international congresses, and to designate the time and place for holding them. The organization of the international movement is thus planned and assured upon systematic lines, and it is at St. Louis, in America, that the first period of organized activity of this great power in dentistry will be terminated and the second period inaugurated.

In all probability it will be many years before such a notable meeting will again be accessible to American practitioners in their own country. We are on the eve of perhaps the greatest event in the history of the world's dentistry. Every professional consideration, every purely selfish interest, alike demands that we should take an active part in this congress. Let the man who claims that American dentistry leads the world go to St. Louis and help to substantiate that claim next month or else hereafter forever hold his peace. America has issued the invitation, you have taken your part in confirming the action of our national association in asking the dental representatives of the nations of the earth to be your guests at St. Louis. The committee has done its work, the feast is prepared, the guests are even now arriving. Let us one and all embrace the opportunity to show them in the best sense our qualities as hosts, and at the same time learn something in a practical way of the good which must accrue to our profession from the development of the international idea, and then give it practical furtherance by our individual efforts.

